# Doping Effects on Magnetic and Electronic Transport Properties in (Ba_1−x_Rb_x_)(Zn_1−y_Mn_y_)_2_As_2_ (0.1 ≤ x, y ≤ 0.25)

**DOI:** 10.3390/nano15130975

**Published:** 2025-06-23

**Authors:** Guoqiang Zhao, Yi Peng, Kenji M. Kojima, Yipeng Cai, Xiang Li, Kan Zhao, Shengli Guo, Wei Han, Yongqing Li, Fanlong Ning, Xiancheng Wang, Bo Gu, Gang Su, Sadamichi Maekawa, Yasutomo J. Uemura, Changqing Jin

**Affiliations:** 1 Kavli Institute for Theoretical Sciences (KITS), University of Chinese Academy of Sciences, Beijing 101408, China; g.q.zhao@iphy.ac.cn (G.Z.); lixiang21@mails.ucas.ac.cn (X.L.); sugang@itp.ac.cn (G.S.); sadamichi.maekawa@riken.jp (S.M.); 2 Beijing National Laboratory for Condensed Matter Physics, Institute of Physics, Chinese Academy of Sciences, Beijing 100190, China; ypeng@iphy.ac.cn (Y.P.); zhaokan1986@163.com (K.Z.); yqli@iphy.ac.cn (Y.L.); wangxiancheng@aphy.iphy.ac.cn (X.W.); 3 Department of Physics, Columbia University, New York, NY 10027, USA; caidia52@gmail.com; 4 TRIUMF, Vancouver, BC V6T 2A3, Canada; kojima@triumf.ca; 5 School of Physics, University of Chinese Academy of Sciences, Beijing 101408, China; 6 School of Physics, Zhejiang University, Hangzhou 310027, China; slguo@zju.edu.cn (S.G.); ningfl@zju.edu.cn (F.N.); 7 Collaborative Innovation Center of Advanced Microstructures, Nanjing University, Nanjing 210093, China; 8 School of Physics and Electronic Engineering, Hebei Minzu Normal University, Chengde 067000, China; whan2013@hbun.edu.cn; 9 Institute of Theoretical Physics, Chinese Academy of Sciences, Beijing 100190, China; 10 RIKEN Center for Emergent Matter Science (CEMS), Wako 315-0198, Japan; 11 Advanced Science Research Center (ASRC), Japan Atomic Energy Agency, Tokai 319-1195, Japan

**Keywords:** diluted magnetic semiconductor, colossal magnetoresistance, Curie temperature, carrier concentration, homogeneous ferromagnets

## Abstract

Diluted magnetic semiconductors (DMSs) represent a significant area of interest for research and applications in spintronics. Recently, DMSs derived from BaZn_2_As_2_ have garnered significant interest due to the record Curie temperature (*T*_C_) of 260 K. However, the influence of doping on their magnetic evolution and transport characteristics has not been thoroughly investigated. This study aims to fill this gap through susceptibility and magnetization measurements, electric transport analysis, and muon spin relaxation and rotation (µSR) measurements on (Ba_1−x_Rb_x_)(Zn_1−y_Mn_y_)_2_As_2_ (0.1 ≤ x, y ≤ 0.25, BRZMA). Key findings include the following: (1) BRZMA showed a maximum *T*_C_ of 138 K, much lower than (Ba,K)(Zn,Mn)_2_As, because of a reduced carrier concentration. (2) A substantial electromagnetic coupling is evidenced by a negative magnetoresistance of up to 34% observed in optimally doped BRZMA. (3) A 100% static magnetic ordered volume fraction is achieved in the low-temperature region, indicating a homogeneous magnet. (4) Furthermore, a systematic and innovative methodology has been initially proposed, characterized by clear step-by-step instructions aimed at enhancing *T*_C_, grounded in robust experimental findings. The findings presented provide valuable insights into the spin–charge interplay concerning magnetic and electronic transport properties. Furthermore, they offer clear direction for the investigation of higher *T*_C_ DMSs.

## 1. Introduction

Diluted magnetic semiconductors (DMSs) [1,2,3,4] are crucial for studying the complex interactions between magnetism and transport properties, with significant potential for spintronic applications [5,6,7]. Among them [3], diluted ferromagnetic semiconductors (DFSs), such as (Ga,Mn)As and its related III–V compounds, have been extensively researched [2,7,8,9] since their initial discovery [10] and subsequent advancements [11]. However, their fabrication into thin films is limited to low-temperature molecular beam epitaxy (LT-MBE) [2], restricting access to key physical measurements, such as neutron scattering for magnetic structure detection [6]. Furthermore, controlling charge and spin concentration doping separately in manganese (Mn)-doped III-V DFSs is difficult due to the partial substitution of trivalent gallium (Ga^3+^) with divalent Mn^2+^. This limitation hinders comprehensive investigations into individual carriers and spin doping effects, intensifying the debate over microscopic description of magnetic interactions [8,12].

Since 2011, a new class of DFSs with independent spin and charge doping mechanisms has emerged [13], confirming theoretical predictions [14], and driving substantial experimental research growth [4]. Among these new material systems, BaZn_2_As_2_ (BZA)-based DFSs have garnered significant attention due to their exceptionally high Curie temperature (*T*_C_) of 260 K [15], and their exotic properties [16,17,18,19,20,21,22,23,24,25,26,27,28,29,30,31]. Previous research shows that the parent compound BaZn_2_As_2_ crystallizes in the ThCr_2_Si_2_-type structure at high temperatures exceeding 900 °C, which is characterized by the space group I4/mmm [16]. This compound is a non-magnetic semiconductor with a bandgap of approximately 0.2 eV, in which carrier and spin co-doping could indue ferromagnetism [16,32,33,34,35,36]. The valence states of barium (Ba), zinc (Zn), and arsenic (As) are +2, +2, and −3, respectively, to attain electrochemical equilibrium. Although manganese (Mn) is a multi-valent ion, its valence in BZA DFSs is 2+, as confirmed by the Mn *L*_2_,_3_-edge X-ray absorption spectroscopy (XAS) (see figure 2 in Ref. [19]). Consequently, carriers can only be introduced through the replacement of Ba^2+^ with K^+^ or Rb^+^. Despite the passage of over a decade since the demonstration of carrier doping effects through Ba^2+^/K^1+^ substitution in (Ba,K)(Zn,Mn)_2_As_2_ (BKZMA) DFSs using magnetization techniques [16], a comprehensive investigation that separately examines the effects of both carrier and spin doping within this material system has yet to be conducted. This study seeks to address this gap for the first time by investigating the newly fabricated polycrystalline specimens (Ba,Rb)(Zn,Mn)_2_As_2_ (BRZMA) DFSs using magnetization, electrical transport, and muon spin rotation/relaxation/resonance (µSR) measurements. More importantly, we also propose a systematic and innovative methodology, characterized by clear step-by-step instructions aimed at enhancing *T*_C_, grounded in robust experimental findings.

## 2. Materials and Methods

Polycrystalline specimens of (Ba_1−x_Rb_x_)(Zn_1−y_Mn_y_)_2_As_2_ (where x = 0.10, 0.15, 0.20, and 0.25; y = 0.10, 0.15, 0.20, and 0.25) were synthesized using a solid-state reaction method [13,16,37] with high-purity reagents produced by Alpha Company. The precursor materials, BaAs and RbAs, were initially sintered at 500 °C and 200 °C for 40 h, respectively, in a sealed titanium tube containing high-purity Ba, rubidium (Rb), and As. These precursors, along with Zn, Mn, and As powders, were loaded into a titanium tube according to the nominal composition of (Ba,Rb)(Zn,Mn)_2_As_2_, under an argon atmosphere at 1 atm pressure, before being placed in a quartz tube. The mixtures were gradually heated to 750 °C at a rate of 3 °C/min and maintained at that temperature for 20 h before cooling to room temperature at a rate of 2 °C/min at the Institute of Physics, Chinese Academy of Siences (IoPCAS) in Beijing, China. The specimens were analyzed using X-ray powder diffraction with a Philips X’pert diffractometer employing Cu Kα radiation at room temperature at the IoPCAS in Beijing China. Direct current (DC) magnetic susceptibility was characterized by using a superconducting quantum interference device (SQUID) magnetometer (Quantum Design), while electrical transport measurements were conducted using a four-probe technique on a physical property measurement system (PPMS, Quantum Design) at the IoPCAS in Beijing China. Positive muon spin relaxation (μSR) measurements were performed on polycrystalline specimens (Ba_0.8_Rb_0.2_)(Zn_0.85_Mn_0.15_)_2_As_2,_ which measure approximately 5 mm in diameter and 6 mm in thickness, at TRIUMF in Vancouver, Canada.

## 3. Results and Discussions

### 3.1. Crystal Structure Characterization

**Figure 1:**
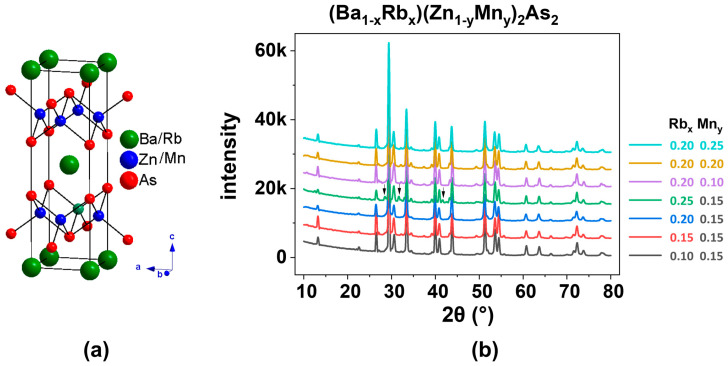
(**a**) The crystal structure of (Ba,Rb)(Zn,Mn)_2_As_2_. (**b**) X-ray diffraction (XRD) pattern of the powder specimen (Ba_1−x_Rb_x_)(Zn_1−y_Mn_y_)_2_As_2_, where x = 0.10, 0.15, 0.20, and 0.25; y = 0.10, 0.15, 0.20 and 0.25. The black arrow indicates the low-temperature BaZn_2_As_2_-based phase, which belongs to the space group *Pnma* [16].

**Figure 2:**
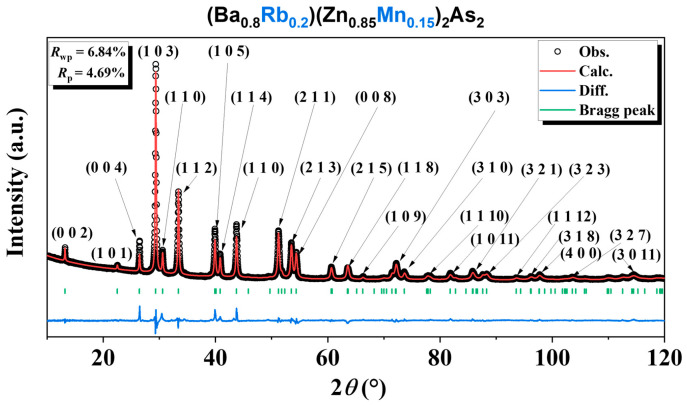
X-ray diffraction pattern from powder specimen of (Ba_0.80_Rb_0.20_)(Zn_0.85_Mn_0.15_)_2_As_2_ with Rietveld analyses.

Like the parent compounds, β-BaZn_2_As_2_ and BKZMA [16], (Ba_1−x_Rb_x_)(Zn_1−y_Mn_y_)_2_As_2_ (where x = 0.10, 0.15, 0.20, and 0.25; y = 0.10, 0.15, 0.20, and 0.25) also crystallizes in the ThCr_2_Si_2_-type structure, as illustrated in Figure 1. This compound features alternating layers of Ba or Rb, and (Zn,Mn)As, formed from edge-sharing ZnAs_4_ tetrahedra, resulting in a quasi-two-dimensional (2D) architecture (Figure 1a). The X-ray diffraction (XRD) patterns (Figure 1b) and the refined data obtained from the powder specimen of (Ba_0.8_Rb_0.2_)(Zn_0.85_Mn_0.15_)_2_As_2_, analyzed through Rietveld refinement (Figure 2), can be accurately indexed to a single phase. The resultant weighted reliability factor (R_wp_) is approximately 6.84%, which shows a reliable analysis.

**Figure 3:**
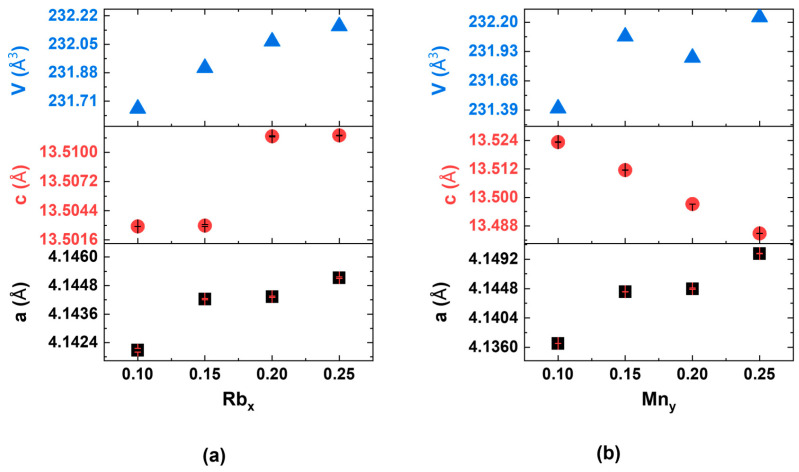
The lattice parameters exhibit a monotonic variation as a function of doping, thereby indicating the successful incorporation of dopants. (**a**) Rb doping in (Ba_1−x_Rb_x_)(Zn_0.85_Mn_0.15_)_2_As_2_ for x = 0.10 (131.3 nm), 0.15 (52.7 nm), 0.20 (429.2 nm), and 0.25 (40.1 nm). (**b**) Mn doping in (Ba_0.8_Rb_0.2_)(Zn_1−y_Mn_y_)_2_As_2_ for y = 0.10 (20.2 nm), 0.15 (429.2 nm), 0.20 (20.7 nm), and 0.25 (447.1 nm). Note that the values in brackets represent the crystallite size obtained from Rietveld refinement. The error bars of the a and c parameters are also indicated.

The larger ionic radius of Rb^+^ (1.52 Å) compared to Ba^2+^ (1.35 Å) results in a monotonic variation in lattice parameters with Ba^2+^/Rb^+^ replacement (Figure 3a), with the coordination numbers of six, consistent with Vegard’s law [38] and confirming successful chemical doping. Vegard’s law for alloyed materials is a linear function: *E*_alloy_ = x*E*_A_ +(1 − x)*E*_B_, where *E*_A_, *E*_B_, and *E*_alloy_ are the respective properties of pure A, Pure B, and the alloy *A*_x_*B*_1−x_, and x is the fraction of one ingredient in a materials point. The replacement of Zn^2+^/Mn^2+^ magnetic ions shows distinct behaviors, as illustrated in Figure 3b. The unit cell volume increases due to the smaller ionic radius of Zn^2+^ (0.60 Å) compared to Mn^2+^ (0.66 Å), with the coordination numbers of four. However, there was an increase in the a-axis and a decrease in the c-axis. It is important to note that the length of the c-axis is approximately three times greater than that of the a-axis. We infer that this discrepancy is due to the distortion of the (Zn,Mn)As_4_ tetrahedra, which results from the combined effects of differing ionic radii and magnetic coupling. A similar trend was noted in the BZA-based DFSs (see figure 1b in Ref. [17]).

### 3.2. Magnetic and Electronic Transport Properties

**Figure 4:**
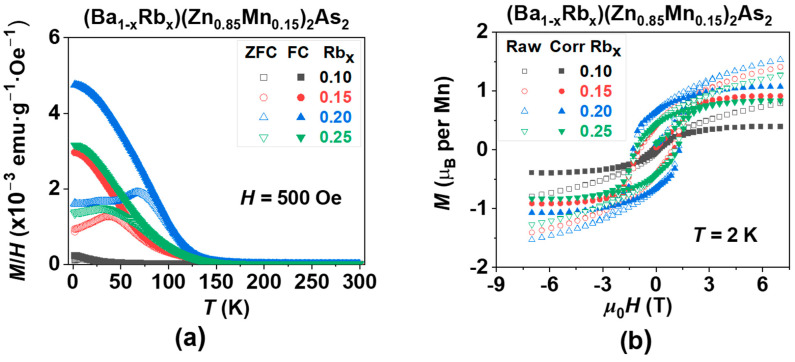
The effect of carrier doping on the magnetization of (Ba_1−x_Rb_x_)(Zn_0.85_Mn_0.15_)_2_As_2_ (where x = 0.10, 0.15, 0.20, and 0.25). (**a**) The direct current (DC) magnetic susceptibility was assessed in a magnetic field of *H* = 500 Gauss (Oe). (**b**) The magnetic hysteresis curve *M*(*H*) was obtained through field training, along with the subtraction of the paramagnetic component, measured at a temperature of *T* = 2K.

Under an external magnetic field of 500 Gauss (Oe), the magnetic susceptibility measured during zero-field-cooled (ZFC, represented by empty symbols) and field-cooled (FC, represented by solid symbols) for (Ba_1−x_Rb_x_)(Zn_0.85_Mn_0.15_)_2_As_2_ (where x = 0.10, 0.15, 0.20, and 0.25) is illustrated in Figure 4. It is widely acknowledged that the ferromagnetic ordering temperature, commonly referred to as the *T*_C_, is associated with the transition of spontaneous magnetization from a value of zero to a non-zero state. This transition reflects the magnitude of the exchange integral from a microscopic perspective.

**Figure 5:**
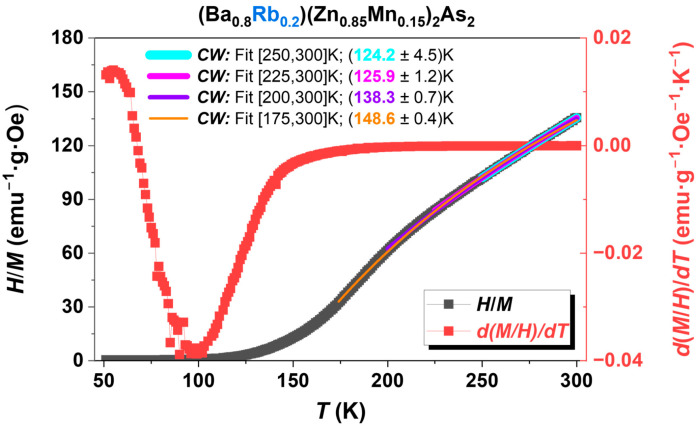
The temperature dependence of the inverse susceptibility of the polycrystalline sample (Ba_0.8_Rb_0.2_)(Zn_0.85_Mn_0.15_)_2_As_2_ is shown (black line). The analysis of the *T*_C_ was conducted utilizing a modified form of the Curie–Weiss law [39], across different temperature regions for comparative analysis. Furthermore, a first-order analysis of magnetic susceptibility, *d*(*M*/*H*)/*dT*, was conducted to elucidate the broad transitions from ferromagnetic to paramagnetic states (red line).

**Figure 11:**
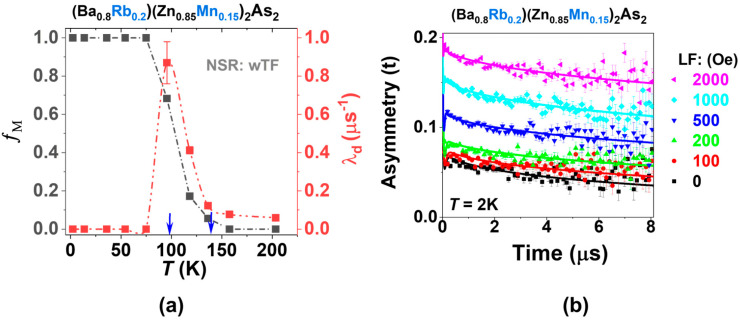
(**a**) Analyzed results of (Ba_0.8_Rb_0.2_)(Zn_1−y_Mn_y_)_2_As_2_, obtained from the wTF spectrum. Static ordered volume fraction, *f*_M_, and the relaxation rate, λ_d_, as functions of temperature. The blue rows indicate the *T*_C_, which is derived from the Curie–Weiss law and the *d*(*M*/*H*)/*dT*, respectively. (**b**) Longitudinal field (LF)–μSR spectra of (Ba_0.8_Rb_0.2_)(Zn_1−y_Mn_y_)_2_As_2_ were measured at a base temperature of *T* = 2 K.

The Curie–Weiss law represents one of the most straightforward methodologies for determining the value of *T*_C_ [39], as evidenced in various similar DMS materials with the independent spin and charge doping mechanism [13,16,17,26,30,37,40,41,42,43,44,45,46,47,48,49,50,51,52,53,54,55,56,57,58]. Following the same idea, we also employed a modified Curie–Weiss law [39] to ascertain the *T*_C_, as detailed below.

$$\chi =\frac{C}{T-\theta_{cw}}+\chi_{0}$$

where *C* is the Curie constant, and θ_cw_ refers to the Curie–Weiss temperature or *T*_C_. Compared to the typical Curie–Weiss law, a temperature-independent small positive susceptibility ($\chi_{0}$) was added. This may be attributed to Pauli paramagnetism [39], originating from the non-localized conduction electrons via Ba^2+^/Rb^1+^. The sample (Ba_0.8_Rb_0.2_)(Zn_0.85_Mn_0.15_)_2_As_2_, illustrated in Figure 5, serves as a representative example. In DMSs, the transition from paramagnetic to ferromagnetic states generally occurs over a relatively wide range of temperature regions [1,6,7]. A comparable phenomenon is clearly observable in the first-order analysis of magnetic susceptibility for BRZMA, particularly in the derivative *d*(*M*/*H*)/*dT*, as illustrated in Figure 5 (red line). Significant changes are observed starting at approximately 150 K, reaching a maximum around 99 K, which strongly indicates that the transition center, *T*_C_, is unequivocally situated within this region. It can be stated that we are able to ascertain relatively accurate values by employing optimized analysis temperature regions in conjunction with the modified Curie–Weiss law [39]. As shown in Figure 5, a maximum value of approximately 149 K and a minimum value of 124 K were obtained across different temperature analysis regions. We chose the fitting temperature region from 200 K to 300 K to determine the *T*_C_ with the value of approximately 138 K. This value is further substantiated by the subsequent results from muon spin rotation (µSR; see Figure 11a presented in the section on static and dynamic magnetism). Briefly speaking, the static ordered volume fraction, *f*_M_, generally begins to increase near the transition temperatures of magnetic materials and ultimately attains 100% in homogeneous magnets at a sufficiently low temperature [59]. In the BRZMA system, with a Rb value of 20% and a Mn value of 15%, *f*_M_ demonstrates a variation from 6% at 136 K to 17% at 118 K, which corresponds precisely with the estimated critical temperature (*T*_C_) of 138 K. In this respect, more insight will be given in the following sections.

**Figure 6:**
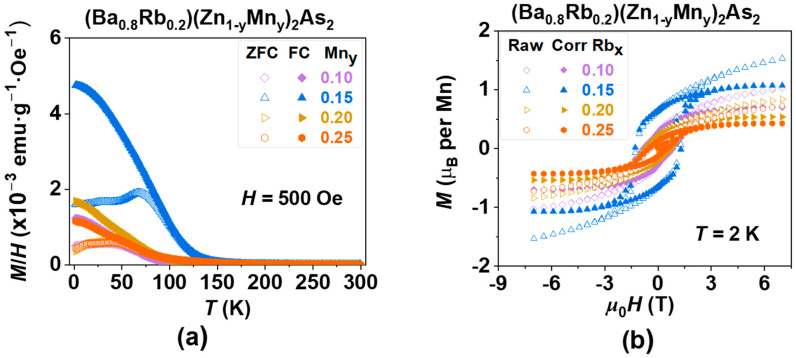
The effect of spin doping on the magnetization of (Ba_0.8_Rb_0.2_)(Zn_1−y_Mn_y_)_2_As_2_ (where y = 0.10, 0.15, 0.20, and 0.25). (**a**) DC magnetic susceptibility was assessed in a magnetic field of *H* = 500 Oe. (**b**) The magnetic hysteresis curve, *M*(*H*), was obtained through field training, along with the subtraction of the paramagnetic component, measured at a temperature of *T* = 2 K.

For a comparable analysis, we utilized the same fitting regions ranging from 200 K to 300 K to ascertain all values of *T*_C_ in (Ba_1−x_Rb_x_)(Zn_1−y_Mn_y_)_2_As_2_ (0.1 ≤ x, y ≤ 0.25), as illustrated in Figure 4a and Figure 6a. The values presented are subsequently summarized in Table 1. An initial increase in the doping levels of Rb or Mn results in a corresponding increase in *T*_C_, which is followed by a decrease, suggesting the existence of an optimal doping concentration. Furthermore, the optimal doping levels of Rb at 20% and Mn at 15% can also be characterized by the maximum values of both the coercive force (*H*_C_ = 1.29 T) and the magnetic remanence (*M*_r_ = 1.07 µB), as illustrated in Figure 4b and Figure 6b, which are also summarized in Table 1.

**Figure 7:**
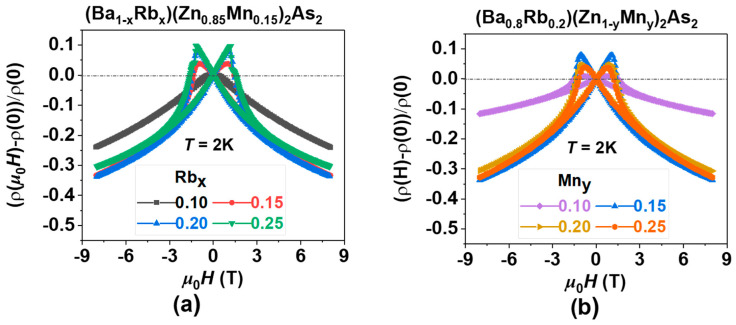
The negative magnetoresistance (MR) is presented as a function of the external magnetic field, measured at a temperature of *T* = 2 K. (**a**) (Ba_1−x_Rb_x_)(Zn_0.85_Mn_0.15_)_2_As_2_ (where x = 0.10, 0.15, 0.20, and 0.25). (**b**) (Ba_0.8_Rb_0.2_)(Zn_1−y_Mn_y_)_2_As_2_ (where y = 0.10, 0.15, 0.20, and 0.25).

Figure 7 presents the magnetoresistance (MR) in BRZMA, where MR is defined as MR = [ρ(*μ*_0_*H*) − ρ(0)]/ρ(0). Though various DMSs exhibit this characteristic behavior [8,60,61], the underlying physics of MR becomes increasingly intricate due to the interplay of multiple degrees of freedom [62,63], which include spin, charge, orbital, and lattice dynamics, in addition to factors such as disorder and strong electron correlation. Nonetheless, it is evident that doping effects can effectively tune the MR in BRZMA, as illustrated in Figure 7a,b. As the levels of Rb doping increase, the MR value initially exhibits an increase, followed by a subsequent decrease. A comparable trend in MR values was noted with escalating levels of Mn doping. In conclusion, the MR value reaches its maximum of approximately 34% at the optimal doping levels of Rb and Mn, as summarized in Table 1. Recent investigations into Na(Zn,Mn)Sb [64,65] have clarified the MR effects through modifications in the band structure, both experimentally and theoretically. We infer that a similar explanation may also occur in BRZMA. The introduction of hole carriers by Ba^2+^/Rb^1+^ modulates the Fermi level, while Zn^2+^/Mn^2+^ involvement gradually adjusts the spin structures. As temperature decreases, spins align to minimize energy, forming a metastable phase at the *T*_C_. External magnetic fields can realign spins and magnetic domains step by step as strength increases from 0 to 7 T. Scattering effects minimize when all spins align and magnetic domains are in one direction, resembling a uniform FM state, resulting in observable MR effects.

**Figure 8:**
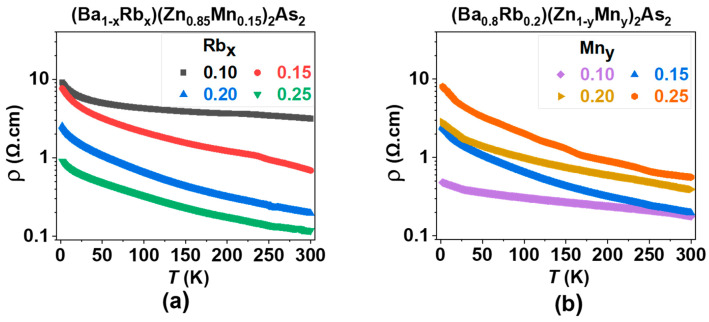
The resistivity is plotted as a function of temperature. (**a**) (Ba_1−x_Rb_x_)(Zn_0.85_Mn_0.15_)_2_As_2_ (where x = 0.10, 0.15, 0.20, and 0.25). (**b**) (Ba_0.8_Rb_0.2_)(Zn_1−y_Mn_y_)_2_As_2_ (where y = 0.10, 0.15, 0.20, and 0.25).

The temperature dependence of resistivity, denoted as ρ(T), demonstrates semiconducting characteristics across all specimens of (Ba_1−x_Rb_x_)(Zn_0.85_Mn_0.15_)_2_As_2_, where x takes values of 0.10, 0.15, 0.20, and 0.25, as illustrated in Figure 8a. An increase in the Rb doping level correlates with a continuous reduction in the magnitude of ρ(T), indicating the effective introduction of hole carriers. It is important to note that the energy gap is approximately 27 meV, as estimated by the thermal activation model for the sample containing 20% Rb and 15% Mn. Semiconducting behavior was observed in all specimens of (Ba_0.8_Rb_0.2_)(Zn_1−y_Mn_y_)_2_As_2_ (where y = 0.10, 0.15, 0.20, and 0.25), as shown in Figure 8b. As the Mn doping levels increase, there is a consistent rise in the magnitude of ρ(T), indicating enhanced scattering effects due to the presence of Mn. Additionally, the specific doping level (x = 0.2, y = 0.15) also yields the highest values for MR, as shown in Figure 7.

### 3.3. Origin of a Lower T_C_

**Figure 9:**
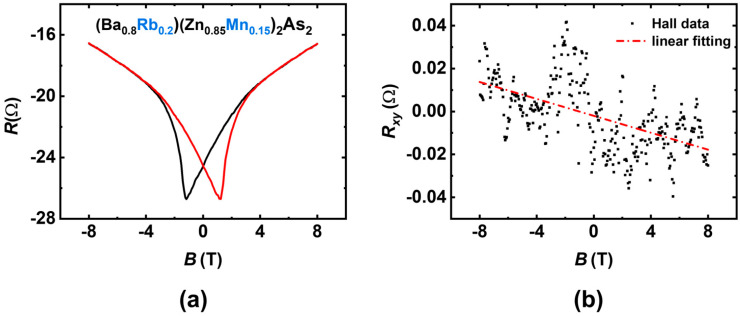
(**a**) The raw data acquired from Hall effect measurement. (**b**) The obtained Hall data (blacked dots) obtained after anti-symmetric treatment, along with the linear-fitting results (red line).

In Table 1, the sample with Rb at 20% and Mn at 15% exhibited a maximum *T*_C_ of 138 K, which is significantly lower than that of BKZMA. Previous research on BKZMA [15] has revealed that a sufficient carrier concentration can suppress the short-range antiferromagnetic interactions between the nearest Mn-Mn pairs, thereby inducing a higher *T*_C_. We infer that the lower carrier concentration in BRMZA results in a lower *T*_C_. Consequently, we have measured the Hall effect in the sample with Rb at 20% and Mn at 15% using a homemade Hall bar structure, as shown in Figure 9. The raw data are shown in Figure 9a, which contains a significant longitudinal resistance signal resulting from electrode asymmetry. To extract the genuine Hall signal, we performed an antisymmetric process as follows:

$$R_{xy}^{+}\left(B\right)=\frac{R^{+}\left(B\right)-R^{-}\left(-B\right)}{2}$$

$$R_{xy}^{-}\left(B\right)=\frac{R^{-}\left(B\right)-R^{+}\left(-B\right)}{2}$$

where $R^{+}\left(B\right)$ and $R^{-}\left(B\right)$ represent the magnetoresistance measured under forward and reverse magnetic field orientations, respectively. Owing to magnetic hysteresis, $R^{+}\left(B\right)$ and $R^{-}\left(B\right)$ exhibit non-overlapping behavior, as illustrated in Figure 9a. The Hall coefficients (R_H_), derived from linear fitting of $R_{xy}^{+}\left(B\right)$ and $R_{xy}^{-}\left(B\right)$, are consistent, from which we can get the electron carrier density, $n=\frac{1}{R_{H}et}=7.14\times 10^{18}\;{cm}^{-3}$, where *t* = 0.44 mm is the sample thickness. It should be noted that, in the calculations, we assume a uniform sample thickness and homogeneous current distribution. However, these assumptions are highly idealized for polycrystalline samples, and, thus, the carrier densities can only be estimated to be within an order of magnitude of 10^19^ cm^−3^. The summarized results for BRZMA and BKZMA are presented in Table 2, which confirms our hypothesis that lower carrier concentrations lead to a decrease in *T*_C_. The ionic radii of K^+^ (1.38 Å) and Ba^2+^ (1.35 Å) are quite similar; consequently, the carrier can be successfully introduced via K^+^/Ba^2+^ replacement with minimal disruption to the crystal structure. However, the radius of Rb^+^ (1.52 Å) is significantly larger than that of Ba^2+^, making carrier doping considerably more challenging from a crystallographic perspective. In addition, the nominal doping ratio is also different. This may account for the low carrier concentration in the BRZMA system, compared to BKZMA system, despite having the same nominal doping levels.

### 3.4. Solution for a Higher T_C_

According to the results of BRZMA, we know that there exist optimal doping levels for carrier and spin concentrations. Consequently, we can tune the magnetism to achieve a higher *T*_C_ through the following methods in both the exploration of new materials and the modification of the properties of existing materials. Let us consider BKZMA as a case study, as it holds a record *T*_C_ of 260 K [15].

Step 1: Estimation of the approximate optimal spin doping level, denoted as Mn_y_. Fixing the Ba^2+^/K^1+^ carrier doping level at a specific sufficient value, such as 35%, enables significant variation in Zn^2+^/Mn^2+^ replacement, which aids in estimating an optimal spin doping level. This is due to a competition between the Mn-Mn antiferromagnetic interaction and the carrier-induced ferromagnetic interactions [32,33,34,35,36]. Increased Mn doping enhances antiferromagnetic interactions; consequently, the maximum T_C_ occurs with an increase in the Mn doping level.

Step 2: Estimation of the approximate optimal carrier doping level, denoted as K_x_. Fixing the spin concentration with the value of Mn_y_ then enables significant variation in Ba^2+^/K^1^ replacement. In addition to the current studied BRZMA case, it is worth noting that the phenomenon of the optimal level of carrier occurs in various DMSs, such as Li(Zn,Mn)P [40,41] and (Sr,Na)(Zn,Mn)_2_As_2_ [67].

Step 3: Ascertain the optimal carrier doping level, K_max_ and Mn_max_. It is crucial that there exists a close interplay between the carrier concentration and spin. Consequently, K_x_ and Mn_y_ are both independent and interconnected, and they do not correspond to the K_max_ and Mn_max_. Therefore, further fine-tuning of doping levels for K and Mn is necessary to achieve the maximum *T*_C_.

Step 4: Involving chemical pressure. The T_C_ decreases in BZA-based DMSs when physical pressure is applied [21,22,28]. This decrease is attributed to modifications in the magnetic interactions that result from the changes in the configuration of the (Zn,Mn)As_4_ tetrahedra and the strength of interlayer As-As bonding [28]. To be specific, the interlayer As-As distance decreases, while the two As-Zn/Mn-As bond angles change monotonically before the transition of the crystal structure [68]. Furthermore, attempts were also conducted at the As site utilizing chemical pressure [30]; however, no results were obtained at the Ba site. Chemical pressure through equivalent substitution [30] is somehow akin to the physical pressure. In (Sr,Na)(Cd,Mn)_2_As_2_ DFSs [56], the numerical equivalence relationship between the physical pressure and chemical pressure can even be estimated. Consequently, we could use a small amount of Rb^1+^ to replace the K^1+^ in the optimal BKZMA system. Since the radius of Rb^1+^ (1.52 Å) is bigger than that for K^1+^ (1.38 Å) and Ba^2+^ (1.35 Å), this replacement could be viewed as an anti-physical pressure effect and, consequently, can increase T_C_ further. We also suggest that Na^1+^ (1.02 Å) and Rb^1+^ could be used together to finely tune the doping effects to achieve optimal results.

Step 5: Just like the (Ga,Mn)As system, post-annealing treatment [6,8,9,11] may also be needed to further enhance the *T*_C_.

In summary, to achieve a higher *T*_C_, we should first determine the optimal carrier doping levels and then apply chemical pressure through equivalent substitution, together with the post-annealing treatment. However, this strategy may be more effective in single crystals with few defects than in polycrystalline samples when solely considering magnetic interactions. It is worth mentioning that the record 260 K [15] in BKZMA is not the ultimate goal.

### 3.5. Static and Dynamic Magnetism

**Figure 10:**
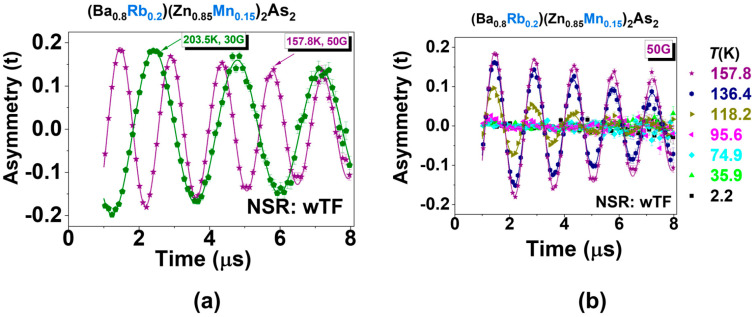
Weak transverse field (wTF)–μSR spectra on the polycrystalline specimen (Ba_0.8_Rb_0.2_)(Zn_1−y_Mn_y_)_2_As_2_. (**a**) The asymmetry attains its maximum value when the temperature surpasses 156.8 K. (**b**) μSR time spectra were recorded at a magnetic field of 50 Oe for all temperature points below 157.8 K. It is important to note that the data corresponding to temperatures of 2.2 K, 35.9 K, and 74.9 K coincide at the point where the asymmetry is equal to zero.

*μ*SR is a sensitive technique utilized to detect both static and dynamic magnetism in various magnetic and superconducting systems [59,69]. Pioneering *μ*SR research on DFSs focused on thin films subjected to appropriate heat treatment, which demonstrated a sharp transition at the *T*_C_ with a complete magnetic volume fraction [70]. Inspired by this, the μSR technique was applied to a new class of DFSs featuring independent spin and charge doping mechanisms to investigate their static and dynamic magnetism [13,16,29,41,47,48,52,67,71,72]. To eliminate the possibility of magnetic impurity clusters in (Ba_0.8_Rb_0.2_)(Zn_0.85_Mn_0.10_)_2_As_2_, we employed the most direct weak transverse field (wTF)-μSR measurements. Figure 10 exhibits the time spectrum, which was analyzed using the following function [59].

Asymmetry (t) = Asy_temp._ [cos (*γ_μ_H*_ext_*t* + *θ*)] exp(−λ_d_t)

The muon gyromagnetic ratio, *γ_μ_*, and the external transverse field, *H*_ext_, are fixed values, while the temperature-dependent asymmetry, Asy_temp._, and relaxation rate, λ*_d_*, serve as free parameters. *θ* represents a phase offset with a fixed value, determined by the initial measured conditions. In the paramagnetic state, the Asy_temp._ reaches its maximum value (Asy_max_) at 157.8 K and 203.5 K, as depicted in Figure 10a. As the temperature decreases, the Asy_max_ decreases significantly, as shown in Figure 10b, serving as an indicator of the static ordered volume fraction, *f*_M_. Notably, (1 − Asy_temp._/Asy_max_) represents *f*_M_ and is plotted in Figure 11a. *f*_M_ begins to build up when crossing *T*_C_ and reaches 100% at sufficiently low temperatures (below 75 K). This indicates that intrinsic homogeneous magnetism has been achieved [59]. Additionally, the relaxation rate, λ*_d_*, is completely suppressed at the base temperature (~2 K). Furthermore, longitudinal field (LF)–*μ*SR measurements were also conducted, verifying the magnetic ground state, as shown in Figure 11b. With the increase in the longitudinal fields to ~2 kOe, the majority part of the sepctra was decoupled; this means that at least the static magnetism dominates the magnetic ground state [59]. With the increase in the longitudinal fields to approximately 2 kOe, the majority of the spectra was decoupled. This indicates that static magnetism predominantly determines the magnetic ground state. Considering the big coercive force (~1.29 T), 100% *f*_M_, and zero λ*_d_*, we can infer that there exists no dynamic magnetism at the base, just similar to the homogeneous magnet Li(Zn,Mn)As [65], in contrast to the SG CuMn [69] and Na(Zn,Mn)Sb [65], or the asperomagnet (Ba,Na)(Zn,Mn)_2_As_2_ [73], in which static and dynamic magnets co-exist. This hypothesis could be tested in the future by increasing the longitudinal field to a higher value, such as 4 kOe.

## 4. Conclusions and Outlook

In summary, a new homogeneous magnet, DFSs BRZMA, in a polycrystalline state was fabricated, achieving a maximum *T*_C_ of 138 K and demonstrating negative magnetoresistance of up to 34%. In comparison to BKZMA, the lower carrier concentration in BRZMA is a key factor contributing to these properties. Consequently, we propose a systematic and novel method for achieving a higher *T*_C_, which encompasses both the exploration of new materials and the modification of the properties of existing materials. These findings provide a clear pathway toward achieving a higher *T*_C_ in DFSs and offer critical insights into the ferromagnetic coupling mechanisms in this field.

Furthermore, there exist numerous intriguing topics for further exploration, although these topics fall outside the scope of the current experimental research study. (1) According to the relaxation rate, *λ*_d_, derived from the wTF-μSR, a possible peak is observed in the position where static ordered volume fractions, *f*_M_, changes between 0 and 100%. It is noteworthy that this peak is situated very close to the minimum of the *d*(*M*/*H*)/*dT*. Consequently, it is imperative to undertake two actions. First, 1/*T*_1_ measurements, derived from the temperature dependence of LF-μSR under a fixed field, are necessary for verification. Second, comprehensive theoretical explanations are required to elucidate the critical behaviors observed. (2) A verification of the proposed method is essential, particularly within the BKZMA system, as it contains records of *T*_C_ with a value of 260 K [15]. (3) In addition to these experimental efforts, theoretical simulations, especially advanced high-throughput simulations and machine learning tools, are crucial for enhancing our understanding of the data and accelerating the pace of research.

## Figures and Tables

**Table 1:** Summary of magnetic and electric transport results for (Ba,Rb)(Zn,Mn)As systems. *H*_C_ is the coercivity, while *M_r_* is the magnetic remanence after the subtraction of a small *T*-linear contribution for a comparable analysis, though some doped materials seem not totally saturated. *MR* is the negative magnetoresistance measured at 2 K, with an external magnetic field of up to 7 T.

(Ba_1−x_Rb_x_)(Zn_1−y_Mn_y_)_2_As_2_		*T*_C_ (K)	*H*_C_ (Oe)	*M*_r_ (μ_B_ per Mn)	*MR* (7 T, 2 K)
x	y				
0.10	0.15	15.2 ± 1.7	1400	0.40	24%
0.15	0.15	138.3 ± 0.9	9900	0.91	33%
0.20	0.15	138.3 ± 0.7	12,900	1.07	34%
0.25	0.15	105.8 ± 0.7	11,600	0.83	31%
0.20	0.10	80.8 ± 1.0	8400	0.70	12%
0.20	0.20	131.1 ± 0.9	7400	0.54	31%
0.20	0.25	125.3 ± 1.4	5300	0.43	33%

**Table 2:** Summary of the *T*_C_ and carrier concentrations for (Ba,Rb)(Zn,Mn)_2_As_2_ and (Ba,K)(Zn,Mn)_2_As_2_. Please note that we use the same carrier concentration, 4.30 × 10^20^ cm^−3^, for all three 10% (Ba,K) substitution samples referenced in [16], based on the fact that Zn^2+^/Mn^2+^ does not introduce additional carriers.

(Ba_1−x_A_x_)(Zn_1−y_Mn_y_)_2_As_2_			Carrier Concentration (cm^−3^)		*T*_C_ (K)	Reference
A	x	Mn_y_				
Rb	0.20	0.15	7.14 × 10^18^	Measured at 2 K	138	Current work
K	0.10	0.05	4.30 × 10^20^	Measured at 2 K	30	[16]
K	0.10	0.10	4.30 × 10^20^	Measured at 2 K	40	[16]
K	0.10	0.15	4.30 × 10^20^	Measured at 2 K	40	[16]
K	0.30	0.15	8.00 × 10^20^	Measured at 250 K	230	[66]
K	0.30	0.24	3.90 × 10^20^	Measured at 5 K	260	[15]

## Data Availability

Raw μSR data are open in the TRIUMF. For further details, please refer to the website https://musr.ca/mud/runSel.html, accessed on 28 November 2019.
